# Characteristics of Users of the Cook for Your Life Website, an Online Nutrition Resource for Persons Affected by Cancer: Descriptive Study

**DOI:** 10.2196/37212

**Published:** 2022-07-05

**Authors:** Eileen Rillamas-Sun, Liza Schattenkerk, Sofia Cobos, Katherine Ueland, Ann Ogden Gaffney, Heather Greenlee

**Affiliations:** 1 Fred Hutchinson Cancer Center Public Health Sciences Seattle, WA United States; 2 Cook for Your Life New York, NY United States

**Keywords:** oncology nutrition, eHealth, website use, bilingual

## Abstract

**Background:**

Accessible nutrition resources tailored to patients with cancer, caregivers of cancer survivors, and people interested in cancer prevention are limited. Cook for Your Life is a bilingual (ie, English and Spanish) website providing science-based, nutrition information for people affected by cancer.

**Objective:**

The aim of this study was to describe the characteristics of Cook for Your Life website users.

**Methods:**

In December 2020, Cook for Your Life website visitors at least 18 years old were invited to participate in an online English-language survey. A Spanish version was offered in April 2021. Demographic, health, and cooking characteristics were collected. Persons with a cancer history were asked about treatment and side effects. Data were analyzed through December 2021 on those completing over half of the survey. Three groups were compared: people with a history of cancer diagnosis, caregivers of cancer survivors, and the general public (ie, people without a cancer history). Website use data were also compared.

**Results:**

Among English-language respondents, 3346 initiated the survey and 2665 (79.65%) completed over half of the questions. Of these, 54.82% (n=1461) had a cancer diagnosis, 8.26% (n=220) were caregivers, and 36.92% (n=984) were from the general public. English-language respondents were US residents (n=2054, 77.07%), with some from Europe (n=285, 10.69%) and Canada (n=170, 6.38%). Cancer survivors were most likely 55 years of age or older, female, non-Hispanic White, with incomes over US $100,000, and college educated. Caregivers and the general public were younger and more racially and geographically diverse. The most common cancer malignancies among English-language cancer survivors were breast (629/1394, 45.12%) and gastrointestinal (209/1394, 14.99%). For Spanish-language respondents, 942 initiated the survey; of these, 681 (72.3%) were analyzed. Of the 681 analyzed, 13.5% (n=92) were cancer survivors, 6.8% (n=46) were caregivers, and 79.7% (n=543) were from the general public. Spanish-language respondents were also more likely to be female and highly educated, but were younger, were from South or Latin America, and had incomes less than US $30,000. Among Spanish-language cancer survivors, breast cancer (27/81, 33%) and gastrointestinal cancer (15/81, 19%) were the most common diagnoses. Website use data on over 2.2 million users from December 2020 to December 2021 showed that 52.29% of traffic was in English and 43.44% was in Spanish. Compared to survey respondents, a higher proportion of website users were male, younger, and from South or Central America and Europe.

**Conclusions:**

Cook for Your Life website users were demographically, socioeconomically, and geographically diverse, especially English-language respondents without a cancer history and all Spanish-language respondents. Improvements on website user diversity and reach for all patients with cancer and research on effective strategies for using this digital platform to support cancer prevention, treatment, and survivorship will continue.

**Trial Registration:**

ClinicalTrials.gov NCT04200482; https://www.clinicaltrials.gov/ct2/show/NCT04200482

## Introduction

Access to science-based nutrition information is vital for all persons affected by cancer, which includes those with a cancer diagnosis, caregivers of patients with cancer and survivors, and those interested in improving their diets for cancer prevention. For patients with cancer who are undergoing treatment, healthy nutrition is an important factor for maintaining energy, improving treatment tolerance and response, reducing cancer symptoms, and addressing the side effects of cancer treatments [1-5]. For cancer survivors, eating a nutritious diet may lower the risk of cancer recurrence and the development of other comorbid diseases, and may help to improve and maintain a high quality of life [6,7]. Patients with cancer and their caregivers have expressed the need to improve their nutrition knowledge, increase self-efficacy about optimal nutrition care, and have greater access to nutrition support [8-10]. Generally, most people are motivated to eat a nutritious diet to reduce the risk of developing many chronic diseases, including cancer, and to improve their overall health.

However, accessible, evidence-based dietary and nutrition resources tailored to the needs of people affected by cancer are limited [11,12]. Indeed, a national survey of 1073 cancer survivors reported that 98% of respondents rated nutrition as important for their cancer care, but only 39% had interacted with a registered dietician [13]. Additionally, a cross-sectional survey conducted among 315 breast cancer survivors reported that 75% used internet searches as their primary source of nutrition advice [14]. This indicates the need for, and importance of, providing clear, relevant, and practical online nutrition education that is backed by sound scientific research.

Cook for Your Life [15] is a website with a broad focus of providing science-based nutrition and culinary education for cancer prevention and for support during cancer treatment and survivorship. Cook for Your Life was acquired by the Fred Hutchinson Cancer Center (Fred Hutch) in 2019 (Textbox 1 [16-18]) and was relaunched in December 2020 with updated features, such as the delivery of bilingual (ie, Spanish and English) videos, recipes, and nutrition education content. Cook for Your Life is funded by Fred Hutch and scientific research grants and does not accept money for advertising or from private or for-profit corporations. Furthermore, all of the Cook for Your Life website content is approved by a board-certified, registered dietician specializing in oncology nutrition, who ensures that all information posted follows the Oncology Nutrition for Clinical Practice guidelines [19].

When the website was relaunched in December 2020, a survey on the English language site was offered to visitors of the website. In April 2021, a Spanish version of the survey was added. The objective of this paper is to describe overall website use and findings from the English and Spanish online user surveys. Improving our knowledge about Cook for Your Life website users is key to understanding how and who uses the website and to determine how effectively we are reaching patients with cancer and survivors, caregivers, and the general population interested in cancer nutrition. With this information, we will be better able to adapt the website content to meet users’ needs and appropriately use the website as a resource and tool in the development of new health interventions to improve cancer prevention, treatment, and survivorship.

**History of the Cook for Your Life website.**

- Cook for Your Life was a nonprofit organization founded in New York City in 2007 by AOG, a three-time cancer survivor. Recognizing that her culinary knowledge helped her better manage the side effects of her chemotherapy treatment, AOG began sharing cooking tips and recipes with other patients with cancer undergoing treatment. These efforts ultimately resulted in free, in-person cooking classes focused on healthy nutrition for patients with cancer and survivors, which were offered through New York City–based cancer centers and community organizations. One of the classes, “Cocinando Saludable, Viviendo Saludable: Promoviendo las compras, el concinar y comer saludable en los que han sobrevivido el cáncer de seno (Healthy Cooking, Healthy Life: Promoting ways to shop, cook and eat healthy foods among breast cancer survivors),” was tailored for Latina breast cancer survivors.
- In 2009, AOG met HG, a cancer epidemiologist and cancer prevention scientist, when she was a faculty member at Columbia University’s Mailman School of Public Health. With funding from the National Cancer Institute (NCI), they developed AOG’s program into a formal curriculum and tested it in the Cocinar Para Su Salud (Cook for Your Health) study (ClinicalTrials.gov NCT01414062) [16,17]. In 2012, AOG founded the Cook for Your Life website [15], recognizing that sharing tips and recipes online could broaden the reach to patients with cancer and survivors worldwide. Using findings from the Cocinar Para Su Salud study, additional NCI funding was obtained to further develop and test the program and include an online component using the Cook for Your Life website in the Mi Vida Saludable (My Healthy Life) trial (ClinicalTrials.gov NCT02780271) [18]. The website became bilingual (ie, English and Spanish) in 2016.
- In 2017, HG moved her research program to the Fred Hutchinson Cancer Center (formerly, the Fred Hutchinson Cancer Research Center) in Seattle, and upon AOG’s retirement in 2019, the Cook for Your Life website was acquired by the Fred Hutchinson Cancer Center. The website has subsequently been used as a nutrition tool and resource for new trials testing digital mobile health interventions to improve lifestyle behaviors among cancer survivors and individuals at risk of developing cancer, with a focus on underserved communities (ClinicalTrials.gov NCT04081298; ClinicalTrials.gov NCT04200482).

## Methods

### Website Analytics

The Cook for Your Life website was built on a WordPress platform and is hosted on Amazon Web Services servers. Web traffic and use data are available from Google Analytics. Traffic data includes the number of new and returning users and number of page views. Web use data on all users includes metrics such as demographic characteristics, behavior on the site, country of residence, and type of device used (eg, mobile or desktop). Web analytics data can be viewed in real time or over specified date ranges.

### Survey Design and Administration

An online survey with questions about demographic characteristics, health and cooking behaviors, and cancer history was created. Usability testing by study staff was conducted to check the survey branching logic and gauge length of time to complete. The English-language version of the survey was released on December 10, 2020, the same day the Cook for Your Life website was relaunched by Fred Hutch. The survey was released in Spanish on April 15, 2021. As an incentive, survey respondents received a customized, downloadable nutrition e-book upon survey completion, either in English or Spanish. Respondents were required to self-report being at least 18 years of age to participate. The Spanish version of the survey was identical to the English version in content and recruitment for participation.

Public-facing visitors to the website were invited to complete the survey through three routes. The first was via a “Volunteer to participate in a research study” link on the home page, the second was a “Get Involved” link in the global navigation at the top, and the third was through a pop-up window appearing to new visitors after 45 seconds on the website. The pop-up window invited users to click a link to participate in a short survey. In addition, the online survey and the request for volunteers was promoted weekly in the website’s digital newsletter, which has a mailing list of approximately 15,000 individuals.

### Ethical Considerations

A waiver of consent was submitted and approved (IRB file number 10567) by the Fred Hutch Institutional Review Board. The survey is administered using the Qualtrics Health Insurance Portability and Accountability Act–secure platform. The survey was completely voluntary. Although lengthy, respondents always had the option to stop answering survey questions at any time by closing the online window. This study was registered at ClinicalTrials.gov (NCT04200482).

### Measures

Web traffic information over time was measured using number of page views per month. Website use data included number of users to the site and users’ gender, age range, and country of residence.

The online survey included questions about demographics, health status and behaviors, diet preferences, and cooking behaviors. Demographic characteristics included age group, gender, country of residence, race and ethnicity, education, household income, number of people living in their household, and whether they live in an urban, suburban, or rural area. Health status and behaviors included presence of noncancer medical conditions, smoking and drinking behavior, height and weight, frequency of fruit and vegetable consumption, and frequency of physical activity. Cooking questions included dietary preference, self-evaluation of cooking ability, frequency of cooking and eating out, and challenges of cooking. Survey respondents with a history of cancer were asked a subset of cancer-related questions, including cancer type, age at diagnosis, treatments received, and side effects of treatment.

### Statistical Analysis

Website data from Google Analytics were reviewed for the period from December 10, 2020, to December 13, 2021, to align with the same window of time that the English-language survey data were analyzed. The Spanish-language survey data were analyzed from users who participated in the survey from April 15 to December 13, 2021. Frequencies by gender, age range, and country of residence from the web use data were compared to the survey respondents.

Respondents completing at least 50% of the survey were included in the analysis. Three mutually exclusive groups were created: (1) cancer survivors, including patients undergoing active treatment; (2) primary caregivers of a patient with cancer or a survivor; and (3) members of the general public interested in cancer prevention. Frequency distributions in the characteristics were calculated across these three groups for the total sample and were stratified by gender. Statistically significant differences comparing cancer survivors to primary caregivers and the general public were tested using the Pearson chi-square test. Distributions of cancer-related characteristics among cancer survivor survey respondents stratified by gender were also estimated.

## Results

**Figure 1:**
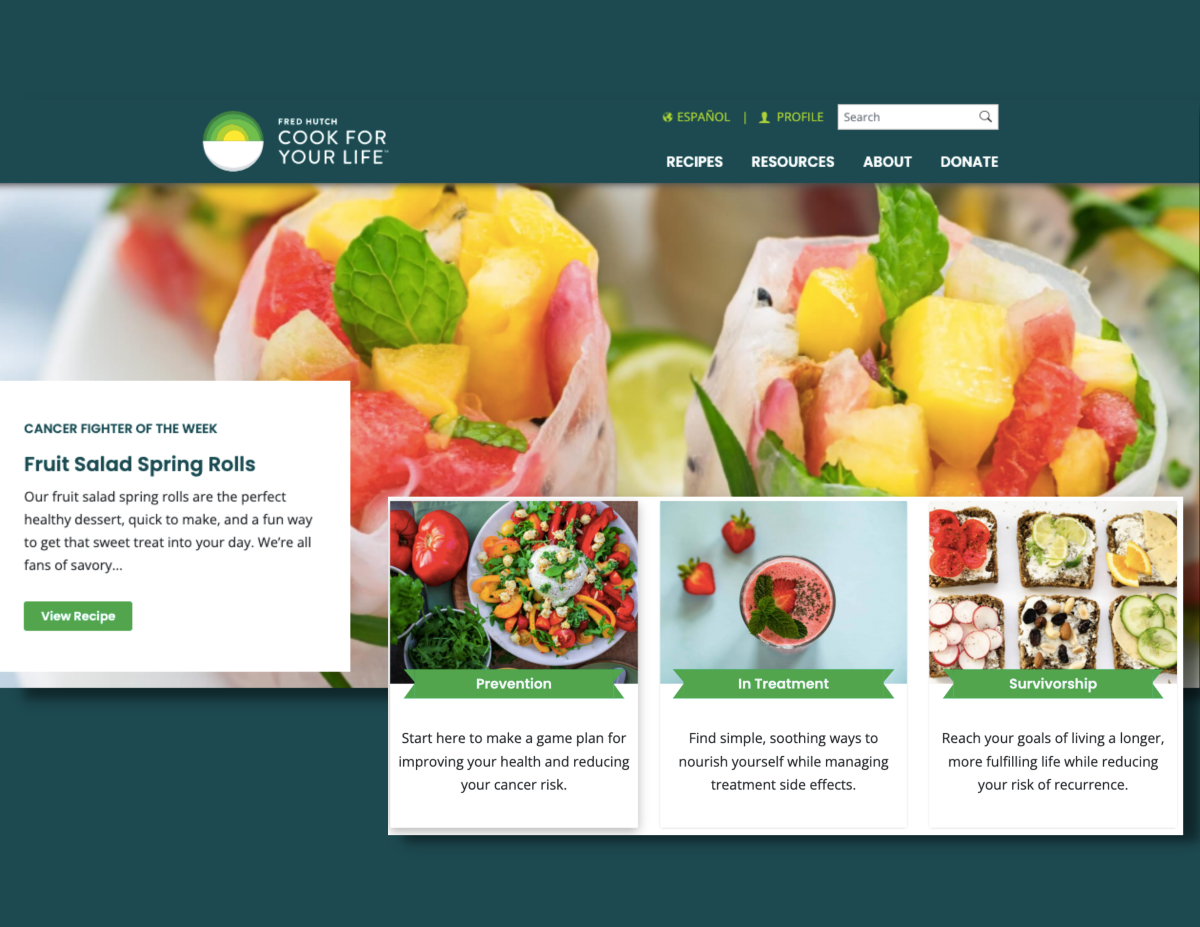
Screenshot of the Cook for Your Life website.

**Figure 2:**
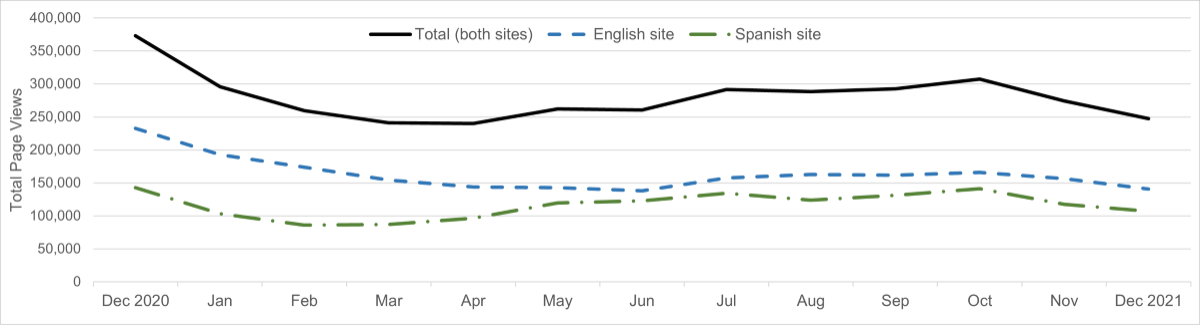
Cook for Your Life website total page views, and page views by English- and Spanish-language website users from December 2020 to December 2021.

From December 10, 2020, to December 13, 2021, a total of 2.08 million unique users visited the Cook for Your Life website (Figure 1). Over this time, there were a total of 3.63 million page views. Monthly total page views for all users and among the English and Spanish sites showed that engagement decreased in the winter months, but increased in spring, stayed level over summer, and began to decrease again by the end of fall (Figure 2). On average, the number of users that visited the site every month was 165,917 (SD 19,668), with the highest number of total users visiting in October 2021 (184,835 users). Use statistics showed that 71.18% of users were female, 38.72% were between 18 and 34 years of age, and 33.15% were between 35 and 54 years of age. Only 12.53% of users were 65 years of age and older. Overall, 30.88% of users were from the United States, 27.07% were from South America, 20.91% were from Europe, and 8.80% were from Central America. Among 1.16 million users who visited the English-language version of the website, most were from the United States (51.63%), the United Kingdom (21.29%), and Canada (9.87%). On the Spanish-language version of the website, out of 1.08 million users, most were from Argentina (25.12%), Spain (14.24%), and Mexico (12.61%).

After 1 year of data collection, 3346 website visitors initiated the English-language survey and, of these, 2665 (79.65%) completed at least 50% the survey. For the Spanish-language survey, 942 visitors initiated the survey, of whom 681 (72.3%) completed at least 50% of the survey. Among all 3346 respondents in either language, 82.79% (n=2770) reported being female, 78.48% (n=2626) were college educated, 78.75% (n=2635) were omnivores, 70.56% (n=2361) rated their cooking skills as intermediate, 62.67% (n=2097) cooked five or more times per week, and 69.04% (n=2310) ate out zero times or one time per week. English-language compared to Spanish-language respondents were more likely to report being a US resident (77.1% vs 11.9%), over 65 years of age (38.3% vs 18.5%), White (80.9% vs 38.0%), non-Hispanic (83.4% vs 5.7%), a nonsmoker (95.7% vs 89.3%), in a household with income over US $60,000 (45.0% vs 13.5%), and engaging in moderate-to-vigorous physical activity (84.9% vs 73.7%). English-language respondents from outside the United States were mainly from Europe (46.6%) or Canada (27.8%), while Spanish-language respondents were more likely to report living in South or Latin America (79.4%).

Characteristics comparing cancer survivors, primary caregivers, and the general public are provided in Tables 1 and 2. The distributions by group for English-language respondents (n=2665) were as follows: 1461 (54.82%) cancer survivors, 220 (8.26%) primary caregivers, and 984 (36.92%) members of the general public. Among English-language respondents overall, cancer survivors compared to either caregivers or the general public were more likely to be US residents, over 65 years of age, non-Hispanic, and nonsmokers, as well as to report eating fruit every day. By gender, demographic characteristics of cancer survivors, caregivers, and the general public for males only and females only were similar in distribution to the overall sample (Tables S1 and S2 in Multimedia Appendix 1). For health characteristics, male cancer survivors were less likely to drink alcohol and to eat over 1 cup of vegetables compared to male caregivers and males from the general public (Table S3 in Multimedia Appendix 1). Female cancer survivors were less likely to be smokers and more likely to eat fruit every day compared to female caregivers and females from the general public (Table S4 in Multimedia Appendix 1).

Of the 681 Spanish-language respondents analyzed, 92 (13.5%) were cancer survivors, 46 (6.8%) were primary caregivers, and 543 (79.7%) were members of the general public. For Spanish-language respondents overall, cancer survivors were more likely to be over 65 years of age and nondrinkers relative to caregivers and members of the general public, but they were generally similar in all other characteristics. By gender, demographic and health characteristics for males across all three groups were similar in distribution to the overall sample of Spanish-language respondents (Tables S1 and S3 in Multimedia Appendix 1). For females, cancer survivors compared to members of the general public were more likely to be over 65 years of age, nonresidents of South or Latin America, hypertensive, and nondrinkers (Tables S2 and S4 in Multimedia Appendix 1).

Clinical characteristics of cancer survivors by gender are shown in Table 3. Among 201 male English-language respondents, the most reported malignancies were gastrointestinal (31.8%) and genitourinary (28.4%). The majority (52.2%) of male patients with cancer were diagnosed after 65 years of age. Over half (n=111) reported being in active cancer treatment, with chemotherapy as the most frequent type of treatment (n=78, 70.3%). Of male patients with cancer in treatment, 87.4% (97/111) reported experiencing side effects in the previous week, the most common being fatigue (72/111, 64.9%), decreased appetite (39/111, 35.1%), and sad feelings (35/111, 31.5%). Among 1177 female English-language respondents, breast cancer was the most reported cancer diagnosis (n=624, 53.02%), followed by gastrointestinal (n=142, 12.06%) and gynecologic (n=124, 10.54%) malignancies. Most (n=477, 40.53%) were diagnosed from 36 to 55 years of age. About half (n=581, 49.36%) reported being in active cancer treatment, with most undergoing chemotherapy (274/581, 47.2%) and endocrine therapy (183/581, 31.5%), and 80.4% (467/581) having had surgery. Of female patients with cancer in treatment, 91.7% (533/581) reported experiencing side effects in the previous week, with the most common being fatigue (373/581, 64.2%), anxiety (201/581, 34.6%), and insomnia (190/581, 32.7%). Among 13 male Spanish-language respondents, the two most common malignancies were gastrointestinal and genitourinary (both 4/13, 31%). Nearly half (6/13, 46%) were diagnosed between 36 and 55 years of age. The majority (11/13, 85%) were in active treatment, and the most common side effects reported in the previous week were anxiety, sad feelings, and general pain (all 4/13, 36%). For the 67 female Spanish-language respondents with cancer history, 40% (27/64) were diagnosed with breast cancer and 48% (32/67) were diagnosed between 36 and 55 years of age. Less than 40% (26/67) were in active treatment, with the most common side effects reported in the past week being fatigue (13/26, 50%), nausea (12/26, 46%), and insomnia (12/26, 46%).

**Table 1:** Frequency of demographic characteristics of Cook for Your Life English- and Spanish-language respondents who completed at least 50% of the online survey.

Characteristic			English respondents (n=2665), n (%)							Spanish respondents (n=681), n (%)				
			Cancer survivors		Primary caregivers	General public			Cancer survivors		Primary caregivers		General public	
Respondents by group			1461 (54.8)		220 (8.3)	984 (36.9)			92 (13.5)		46 (6.8)		543 (79.7)	
**Gender^a,b^**														
	Male	214 (14.7)		18 (8.2)			175 (17.8)	15 (16.3)				11 (23.9)		100 (18.4)
	Female	1230 (84.2)		200 (90.9)			795 (80.8)	76 (82.6)				34 (73.9)		435 (80.1)
**Region of residence^a,b,c^**														
	United States	1206 (82.7)		159 (72.3)			689 (70.2)	15 (16.5)				5 (10.9)		61 (11.3)
	Africa	13 (0.9)		1 (0.5)			12 (1.2)	0 (0)				0 (0)		0 (0)
	Asia or Pacific Islands	42 (2.9)		11 (5.0)			30 (3.1)	1 (1.1)				0 (0)		0 (0)
	Europe	95 (6.5)		23 (10.5)			167 (17.0)	13 (14.3)				5 (10.9)		35 (6.5)
	Middle East	2 (0.1)		1 (0.5)			3 (0.3)	0 (0)				0 (0)		0 (0)
	Canada	84 (5.8)		15 (6.8)			71 (7.2)	0 (0)				0 (0)		3 (0.6)
	South or Latin America	17 (1.2)		10 (4.6)			10 (1.0)	62 (68.1)				36 (78.3)		443 (81.9)
**Age (years)^a,b,c^**														
	18-35	30 (2.1)		19 (8.6)			105 (10.7)	6 (6.5)				8 (17.4)		99 (18.2)
	36-55	391 (26.8)		79 (35.9)			276 (28.1)	35 (38.0)				20 (43.5)		223 (41.1)
	56-65	407 (27.9)		64 (29.1)			273 (27.7)	25 (27.2)				9 (19.6)		130 (23.9)
	66-75	436 (29.8)		49 (22.3)			242 (24.6)	22 (23.9)				7 (15.2)		71 (13.1)
	≥76	197 (13.5)		9 (4.1)			88 (8.9)	4 (4.4)				2 (4.4)		20 (3.7)
**Race^a^**														
	American Indian	9 (0.6)		3 (1.4)			6 (0.6)	2 (2.2)				2 (4.4)		2 (0.4)
	Asian, Native Hawaiian, or Pacific Islander	46 (3.2)		15 (6.8)			37 (3.8)	1 (1.1)				0 (0)		3 (0.6)
	Black or African American	49 (3.4)		10 (4.6)			48 (4.9)	2 (2.2)				0 (0)		5 (0.9)
	White	1216 (83.2)		164 (74.6)			775 (78.8)	37 (40.2)				21 (45.7)		201 (37.0)
	Mixed race	85 (5.8)		17 (7.7)			71 (7.2)	31 (33.7)				17 (37.0)		234 (43.1)
	Other	18 (1.2)		4 (1.8)			14 (1.4)	8 (8.7)				2 (4.4)		45 (8.3)
	Prefer not to say	38 (2.6)		7 (3.2)			33 (3.4)	11 (12.0)				4 (8.7)		53 (9.8)
**Ethnicity^a,b,c^**														
	Hispanic	81 (5.5)		21 (9.6)			60 (6.1)	73 (79.4)				40 (87.0)		480 (88.4)
	Non-Hispanic	1258 (86.1)		177 (80.5)			787 (80.0)	11 (12.0)				2 (4.4)		26 (4.4)
	Prefer not to say	122 (8.4)		22 (10.0)			137 (13.9)	8 (8.7)				4 (8.7)		37 (8.7)
**Education^b^**														
	Less than high school	22 (1.5)		6 (2.7)			35 (3.6)	6 (6.5)				5 (10.9)		40 (7.4)
	High school graduate or GED^d^	111 (7.6)		13 (5.9)			80 (8.1)	13 (14.1)				3 (6.5)		51 (9.4)
	Trade school or associate’s degree	127 (8.7)		21 (9.6)			105 (10.7)	11 (12.0)				5 (10.9)		51 (9.4)
	Some college but not a graduate	211 (14.4)		32 (14.6)			150 (15.2)	14 (15.2)				10 (21.7)		102 (18.8)
	College degree or more	985 (67.4)		147 (66.8)			610 (62.0)	48 (52.2)				23 (50.0)		294 (54.1)
	Other	5 (0.3)		1 (0.5)			4 (0.4)	0 (0)				0 (0)		5 (0.9)
**Household income (US $)^b^**														
	0-30,000	183 (12.5)		38 (17.3)			169 (17.2)	38 (41.3)				21 (45.7)		208 (38.3)
	30,001-60,000	227 (15.5)		38 (17.3)			171 (17.4)	16 (17.4)				5 (10.9)		48 (8.8)
	60,001-100,000	294 (20.1)		44 (20.0)			189 (19.2)	4 (4.4)				3 (6.5)		37 (6.8)
	>$100,000	399 (27.3)		47 (21.4)			225 (22.9)	5 (5.4)				3 (6.5)		40 (7.4)
	Prefer not to say	358 (24.5)		53 (24.1)			230 (23.4)	29 (31.5)				14 (30.4)		210 (38.7)
**Number of people in household^a,b^**														
	1	297 (20.3)		23 (10.5)			232 (23.6)	9 (9.8)				7 (15.2)		60 (11.1)
	2	762 (52.2)		108 (49.1)			428 (43.5)	29 (31.5)				9 (19.6)		155 (28.6)
	3	183 (12.5)		42 (19.1)			125 (12.7)	19 (20.7)				11 (23.9)		124 (22.8)
	≥4	219 (15.0)		47 (21.4)			199 (20.2)	35 (38.0)				19 (41.3)		204 (37.6)
**Area of residence**														
	Urban	455 (31.1)		78 (35.5)			351 (35.7)	64 (69.6)				34 (73.9)		425 (78.3)
	Suburban	691 (47.3)		100 (45.5)			429 (43.6)	22 (23.9)				7 (15.2)		78 (14.4)
	Rural	315 (21.6)		42 (19.1)			204 (20.7)	6 (6.5)				5 (10.9)		40 (7.4)

^a^*P*≤.05 comparing patients with cancer and primary caregivers among English-language survey respondents.

^b^*P*≤.05 comparing patients with cancer and general public among English-language survey respondents.

^c^*P*≤.05 comparing patients with cancer and general public among Spanish-language survey respondents.

^d^GED: General Education Diploma.

**Table 2:** Frequency of health characteristics of Cook for Your Life English- and Spanish-language respondents who completed at least 50% of the online survey.

Characteristic		English respondents (n=2665), n (%)				Spanish respondents (n=681), n (%)		
		Cancer survivors	Primary caregivers	General public	Cancer survivors		Primary caregivers	General public
**Cardiometabolic condition^a^**								
	Chest pain	30 (2.4)	7 (3.4)	23 (2.4)	2 (2.3)		1 (2.4)	20 (3.8)
	Diabetes or prediabetes	200 (14.3)	29 (13.9)	153 (16.0)	16 (18.2)		7 (16.7)	78 (14.8)
	Hypertension	338 (24.2)	55 (26.4)	250 (26.2)	26 (29.6)		9 (21.4)	116 (22.1)
	High cholesterol	290 (20.7)	45 (21.6)	215 (22.5)	18 (20.5)		14 (33.3)	90 (17.1)
	Heart disease	66 (4.7)	10 (4.8)	45 (4.7)	2 (2.3)		3 (7.1)	19 (3.6)
	Vascular disease	37 (2.6)	7 (3.4)	27 (2.8)	2 (2.3)		1 (2.4)	6 (1.1)
Current smoker^a,b^		35 (2.5)	11 (5.3)	64 (6.7)	8 (9.1)		7 (16.7)	55 (10.5)
Drinks alcohol^b,c,d^		625 (45.5)	88 (45.4)	540 (59.0)	22 (25.3)		20 (48.8)	199 (39.2)
**Number of alcoholic drinks^a,b^ (out of those who drink alcohol, as reflected in “Total” row)**								
	Total	619 (100)	86 (100)	535 (100)	22 (100)		20 (100)	195 (100)
	1-2 per week	320 (51.7)	43 (50.0)	204 (38.1)	14 (63.6)		12 (60.0)	133 (68.2)
	3-6 per week	191 (30.9)	23 (26.7)	187 (35.0)	6 (27.3)		6 (30.0)	43 (22.1)
	Every day	57 (9.2)	4 (4.7)	56 (10.5)	1 (4.6)		1 (5.0)	9 (4.6)
	≥2 per day	51 (8.2)	16 (18.6)	88 (16.5)	1 (4.6)		1 (5.0)	10 (5.1)
**Days per week that fruit is eaten^a,b^**								
	None	36 (2.7)	6 (3.1)	37 (4.1)	3 (3.5)		1 (2.4)	16 (3.2)
	1-3 days	255 (18.7)	53 (27.8)	234 (25.8)	24 (27.6)		10 (24.4)	169 (33.6)
	4-6 days	417 (30.6)	64 (33.5)	276 (30.4)	31 (35.6)		17 (41.5)	149 (29.6)
	Every day	653 (48.0)	68 (35.6)	360 (39.7)	29 (33.3)		13 (31.7)	169 (33.6)
**Amount of fruit when eating (out of those who eat fruit, as reflected in “Total” row)**								
	Total	1318 (100)	184 (100)	860 (100)	83 (100)		40 (100)	483 (100)
	<1 cup	853 (64.7)	116 (63.0)	548 (63.7)	50 (60.2)		18 (45.0)	234 (48.5)
	1-2 cups	355 (26.9)	50 (27.2)	235 (27.3)	23 (27.7)		15 (37.5)	176 (36.4)
	>2 cups	110 (8.4)	18 (9.8)	77 (9.0)	10 (12.1)		7 (17.5)	73 (15.1)
**Days per week that vegetables are eaten**								
	None	12 (0.9)	0 (0)	9 (1.0)	2 (2.3)		0 (0)	5 (1.0)
	1-3 days	138 (10.2)	20 (10.5)	103 (11.5)	22 (25.6)		10 (24.4)	123 (24.7)
	4-6 days	420 (31.0)	70 (36.8)	288 (32.1)	32 (37.2)		15 (36.6)	190 (38.1)
	Every day	784 (57.9)	100 (52.6)	497 (55.4)	30 (34.9)		16 (39.0)	181 (36.3)
**Amount of vegetables when eating^a,b^ (out of those who eat vegetables, as reflected in “Total” row) **								
	Total	1321 (100)	184 (100)	849 (100)	84 (100)		41 (100)	473 (100)
	<1 cup	775 (58.7)	95 (51.6)	442 (52.1)	48 (57.1)		23 (56.1)	225 (47.6)
	1-2 cups	437 (33.1)	65 (35.3)	308 (36.3)	27 (32.1)		14 (34.2)	173 (36.6)
	>2 cups	109 (8.3)	24 (13.0)	99 (11.7)	9 (10.7)		4 (9.8)	75 (15.9)
**Days per week of MVPA^e^**								
	None	208 (15.6)	28 (15.2)	123 (14.4)	25 (29.4)		7 (17.1)	127 (26.6)
	1-3 days	475 (35.7)	74 (40.2)	319 (37.3)	35 (41.2)		22 (53.7)	208 (43.5)
	4-6 days	487 (36.6)	62 (33.7)	302 (35.3)	18 (21.2)		11 (26.8)	109 (22.8)
	Every day	162 (12.2)	20 (10.9)	112 (13.1)	7 (8.2)		1 (2.4)	34 (7.1)
**Minutes per day of MVPA^e^**								
	0 to <10	220 (16.5)	31 (16.9)	134 (15.7)	24 (28.2)		6 (14.6)	134 (28.0)
	10 to <30	352 (26.4)	56 (30.4)	214 (25.0)	25 (29.4)		17 (41.5)	110 (23.0)
	30 to <40	296 (22.2)	37 (20.1)	185 (21.6)	17 (20.0)		6 (14.6)	77 (16.1)
	40 to <60	321 (24.1)	41 (22.3)	208 (24.3)	14 (16.5)		9 (22.0)	101 (21.1)
	≥60	143 (10.7)	19 (10.3)	115 (13.4)	5 (5.9)		3 (7.3)	56 (11.7)

^a^*P*≤.05 comparing patients with cancer and primary caregivers among English-language survey respondents.

^b^*P*≤.05 comparing patients with cancer and general public among English-language survey respondents.

^c^*P*≤.05 comparing patients with cancer and primary caregivers among Spanish-language survey respondents.

^d^*P*≤.05 comparing patients with cancer and general public among Spanish-language survey respondents.

^e^MVPA: moderate-to-vigorous physical activity.

**Table 3:** Frequency of cancer-related characteristics of English- and Spanish-language patients with cancer and survivors responding to at least 50% of the Cook for Your Life website survey stratified by gender.

Characteristic			English respondents (n=1394), n (%)				Spanish respondents (n=81), n (%)			
			Male (n=201)		Female (n=1177)		Male (n=13)		Female (n=67)	
**Primary cancer site **										
	Breast	0 (0)		624 (53.0)		0 (0)		27 (40.3)		
	Gastrointestinal region	64 (31.8)		142 (12.1)		4 (30.8)		11 (16.4)		
	Gynecologic region	N/A^a^		124 (10.5)		N/A^a^		10 (14.9)		
	Hematologic region	24 (11.9)		102 (8.7)		0 (0)		1 (1.5)		
	Genitourinary region	57 (28.4)		26 (2.2)		4 (30.8)		3 (4.5)		
	Respiratory region (lung)	10 (5.0)		29 (2.5)		1 (7.7)		3 (4.5)		
	Skin	6 (3.0)		24 (2.0)		1 (7.7)		2 (3.0)		
	Endocrine region (thyroid)	1 (0.5)		20 (1.7)		3 (23.1)		3 (4.5)		
	Other	39 (19.4)		86 (7.3)		0 (0)		7 (10.5)		
**Age at diagnosis (years)**										
	18-35	8 (4.0)		62 (5.3)		1 (7.7)		10 (14.9)		
	36-55	38 (18.9)		477 (40.5)		6 (46.2)		32 (47.8)		
	56-65	50 (24.9)		355 (30.2)		2 (15.4)		16 (23.9)		
	>65	105 (52.2)		283 (24.0)		4 (30.8)		9 (13.4)		
Undergoing treatment			111 (55.2)		581 (49.4)		11 (84.6)		26 (38.8)	
**Type of treatment (out of those undergoing treatment, as reflected in “Total” row)**										
	Total	111 (100)		581 (100)		11 (100)		26 (100)		
	Chemotherapy	78 (70.3)		274 (47.2)		4 (36.4)		13 (50.0)		
	Radiation therapy	24 (21.6)		102 (17.6)		3 (27.3)		5 (19.2)		
	Endocrine therapy	9 (8.1)		183 (31.5)		4 (36.4)		7 (26.9)		
	Targeted therapy or immunotherapy	18 (16.2)		125 (21.5)		0 (0)		2 (7.7)		
	Surgery	64 (57.7)		467 (80.4)		11 (100)		12 (46.2)		
**Side effects in past 7 days^b^ (out of those with side effects, as reflected in “Total” row)**										
	Total	111 (100)		581 (100)		11 (100)		26 (100)		
	Fatigue	72 (64.9)		373 (64.2)		3 (27.3)		13 (50.0)		
	Anxiety	27 (24.3)		201 (34.6)		4 (36.4)		10 (38.5)		
	Insomnia	20 (18.0)		190 (32.7)		2 (18.2)		12 (46.2)		
	Sad feelings	35 (31.5)		169 (29.1)		4 (36.4)		11 (42.3)		
	Nausea	33 (29.7)		159 (27.4)		3 (27.3)		12 (46.2)		
	Dry mouth	24 (21.6)		165 (28.4)		1 (9.1)		9 (34.6)		
	Hot flashes	7 (6.3)		181 (31.2)		2 (18.2)		11 (42.3)		
	Decreased appetite	39 (35.1)		146 (25.1)		3 (27.3)		9 (34.6)		
	General pain	22 (19.8)		151 (26.0)		4 (36.4)		7 (26.9)		
	Taste changes	30 (27.0)		142 (24.4)		1 (9.1)		8 (30.8)		
	Constipation	23 (20.7)		143 (24.6)		2 (18.2)		8 (30.8)		
	Diarrhea	23 (20.7)		130 (22.4)		2 (18.2)		2 (7.7)		
	Mouth sores	6 (5.4)		62 (10.7)		1 (9.1)		3 (11.5)		
	Difficulty swallowing	16 (14.4)		40 (6.9)		1 (9.1)		5 (19.2)		
	Vomiting	9 (8.1)		34 (5.9)		0 (0)		4 (15.4)		

^a^N/A: not applicable for men.

^b^Multiple responses possible.

## Discussion

### Principal Findings

The Cook for Your Life website is a bilingual science-based culinary and nutrition resource for persons affected by cancer, broadly defined as cancer survivors, caregivers of patients with cancer, and people interested in cancer prevention. An important goal of the website is to provide accessible diet and nutrition information to a wide and diverse range of people, including those from underserved populations. To improve our understanding of the website’s reach, we administered a volunteer-based, nonprobability survey on the site. Analysis of these survey data indicated that survey respondents, cancer survivors in particular, were primarily living in the United States and were primarily White, non-Hispanic females who were about 55 years of age or older, highly educated, and of high socioeconomic status. This finding suggests the need to improve the reach and diversity of the Cook for Your Life website user base, especially among patients with cancer and survivors.

Further evaluation of the survey respondents showed that characteristics differed according to whether the survey was taken in English or Spanish. English-language respondents were mainly White, non-Hispanic females with high household incomes and living in the United States. English-language cancer survivors were most likely to fit this description, and most reported having breast cancer. In the United States, breast cancer is the most common malignancy among women, and incidence rates are highest among non-Hispanic White people over 60 years of age [20], which aligns with our own survey respondent profile. Furthermore, a study of nutrition needs among cancer survivors reported that breast cancer survivors expressed the most interest in receiving additional nutrition support [13], while another study found that a large proportion of breast cancer survivors seek nutrition advice online [14]. Motivators for responding to surveys, including online surveys, are knowing who is administering it, the topic area, and the length of time to finish [21]. Referrals to the Cook for Your Life website were primarily from social media or from a doctor or dietician (data not shown), so it is also possible that more women were motivated to respond because of Cook for Your Life’s partnership with Fred Hutch, a well-respected and renowned cancer research organization, along with their own self-interests about cancer and nutrition.

Although cancer survivors were less diverse, English-language respondents without a history of cancer showed more gender, international, racial, and socioeconomic diversity. Spanish-language respondents were also more demographically diverse, including among those reporting a history of cancer. Website use data indicated that Cook for Your Life visitors, in general, included higher proportions of men, were younger (ie, 18-44 years old), and were more globally represented than those who responded to the survey. Furthermore, examination of the cooking questions indicated that many survey respondents cooked for themselves often and regarded their cooking skills to be intermediate, indicating that the Cook for Your Life website is attracting people with already-strong culinary interests. These findings suggest that the content and topic areas of the Cook for Your Life website are appealing to a wide range of people without a history of cancer, and the focus on reach and diversity should be concentrated mainly among patients with cancer and survivors.

While our analysis indicated acceptable demographic, socioeconomic, and geographic diversity and reach among our general users without a history of cancer, the Cook for Your Life website also aims to support nutrition needs of patients with cancer and survivors as well as cancer caregivers. Previous studies have reported that both patients with cancer and their caregivers do not receive adequate nutrition education despite expressing a desire and need for more nutrition support and information [10,11,13]. One of Cook for Your Life’s goals is to be a nutrition resource for patients with cancer and caregivers of patients with cancer undergoing treatment. The literature supports the notion that a healthy diet can improve cancer therapy response and reduce side effects, such as nausea and vomiting, in patients with cancer [1-5]. The survey indicated that most patients with cancer undergoing therapy experienced side effects, which might be improved with nutrition information provided on the Cook for Your Life website. The Cook for Your Life website also serves as a resource and tool for intervention studies aimed at improving health behaviors in patients with cancer and survivors, with a specific focus on underserved populations. This emphasizes the need to ensure that website users with a cancer history are being reached. Therefore, in-depth evaluations focusing on a sampling frame of patients with cancer and survivors may be required to address specific issues, such as content appeal, accessibility, applicability, and usability.

### Limitations

We acknowledge that the survey data represented only those who were agreeable to participating in an online survey and then, among those, who completed at least half of the survey. Web surveys are prone to increased error associated with coverage, sampling, and nonresponse, and those limitations apply to this analysis as well [21,22]. Compared to the respondents of the Spanish-language survey, respondents of the English-language survey were more likely to complete 50% or more of the survey. Also, a higher proportion of patients with cancer and survivors completed the English-language version, and it is likely that their health status was a stronger motivator for greater engagement in the survey. However, it is difficult to know what bias might be introduced in the findings without knowledge about the nonresponders, noncompleters, and those generally not interested in participating in surveys. For web surveys without a clear sampling frame, information about nonresponders and noncompleters cannot be obtained [22]. Coverage error (ie, internet access) is also a study limitation, and studies show that lack of coverage is more likely among non-White racial groups and those of lower socioeconomic status [23], which are the demographic groups that we most need to reach. Lastly, analysis of the race and ethnicity variables indicated that these were US-based constructs, and a large proportion of international respondents were unable to provide answers to these questions. Similarly, the household income response categories were applicable to US respondents and were likely not specific enough for international respondents from countries whose definition of “high” socioeconomic status may be at a cutoff value much lower than that of the United States.

### Comparison With Prior Work

There have been a handful of science-based websites also focused on providing nutrition education and support to patients with cancer and led by cancer research or academic institutions. A publication from a research team in the Netherlands described their process of developing a website titled “Voeding en kanker info” (Nutrition and cancer info) [24], which provides nutrition education for cancer prevention as well as during and after treatment [12]. In addition to their development process, user statistics for the year after their launch (ie, May 2014-May 2015), including total page views, total visitors, and region of user’s residence, were reported; however, details such as demographic, health behavior, and clinical characteristics about their users were not described [12]. A similar project of developing a web-based cookbook for pediatric patients with cancer, undertaken by the MD Anderson Cancer Center in Texas, has also been described [25] and compared to two other science-based cancer and nutrition websites, including Cook for Your Life [26]; however, information about the characteristics of their users was not provided.

### Conclusions

Analysis of users who visited the Cook for Your Life website indicated acceptable demographic, socioeconomic, and geographic reach and diversity for users without a history of cancer in particular. Research to improve our knowledge about the website’s user base, including understanding how people learn and apply knowledge from the website, use of specific website content, and information about website functionality, access, and application, more broadly, will continue. Work focused on improving diversity and reach among patients with cancer and survivors is needed and should include targeting patients with cancer with a clear sampling frame and through various modes of data collection.

## Appendix

Multimedia Appendix 1 Supplementary tables.

## Abbreviations

Fred Hutch: Fred Hutchinson Cancer Center.
