# Supplementary material for: Characteristics of Users of the Cook for Your Life Website, an Online Nutrition Resource for Persons Affected by Cancer: Descriptive Study
Source: JMIR Cancer. 2022 Jul 5;8(3):e37212. doi: 10.2196/37212 (PMC9496813; doi:10.2196/37212)
Supplement: Multimedia Appendix 1 [file cancer_v8i3e37212_app1.docx]

Table S1. Frequency of demographic characteristics of Cook for Your Life English and Spanish male respondents who completed at least 50% of online survey by cancer survivors, primary caregivers, and general public

| Characteristic | English respondents (n=406), n (%) | | | | Spanish Respondents (n=126), n (%) | | | | | |  |
| --- | --- | --- | --- | --- | --- | --- | --- | --- | --- | --- | --- |
|  | Cancer survivors | Primary Caregivers | General public | | Cancer survivors | | Primary caregivers | | General public | |  |
| Respondents by group | 214 (52.6) | 18 (4.4) | 174 (43.0) | | 15 (11.9) | | 11 (8.7) | | 100 (79.4) | |  |
| **Region of residence** |  |  |  | |  | |  | |  | |  |
| United States | 178 (83.2) | 12 (66.7) | 113 (64.9) | | 2 (6.7) | | 2 (18.2) | | 12 (12.0) | |  |
| Africa | 1 (0.5) | 0 (0) | 3 (1.7) | | 0 (0) | | 0 (0) | | 0 (0) | |  |
| Asia or Pacific Islands | 9 (4.2) | 4 (22.2) | 7 (4.0) | | 0 (0) | | 0 (0) | | 0 (0) | |  |
| Europe | 7 (3.3) | 1 (5.6) | 38 (21.8) | | 4 (26.7) | | 2 (18.2) | | 6 (6.0) | |  |
| Middle East | 0 (0) | 0 (0) | 2 (1.2) | | 0 (0) | | 0 (0) | | 0 (0) | |  |
| Canada | 12 (5.6) | 1 (5.6) | 11 (6.3) | | 0 (0) | | 0 (0) | | 0 (0) | |  |
| South or Latin America | 7 (3.3) | 0 (0) | 0 (0) | | 10 (66.7) | | 7 (63.6) | | 82 (82.0) | |  |
| **Age (years)^a^** |  |  |  | |  | |  | |  | |  |
| 18 - 35 | 3 (1.4) | 3 (16.7) | 18 (10.3) | | 0 (0) | | 3 (27.3) | | 10 (10.0) | |  |
| 36 - 55 | 27 (12.6) | 4 (22.2) | 35 (20.0) | | 7 (46.7) | | 4 (36.4) | | 32 (32.0) | |  |
| 56 - 65 | 47 (22.0) | 2 (11.1) | 42 (24.0) | | 1 (6.7) | | 2 (18.2) | | 25 (25.0) | |  |
| 66 - 75 | 79 (36.9) | 8 (44.4) | 57 (32.6) | | 4 (33.3) | | 0 (0) | | 27 (27.0) | |  |
| ≥76 | 58 (27.1) | 1 (5.6) | 23 (13.1) | | 2 (13.3) | | 2 (18.2) | | 6 (6.0) | |  |
| **Race** |  |  |  | |  | |  | |  | |  |
| American Indian | 3 (1.4) | 1 (5.6) | 1 (0.6) | | 0 (0) | | 1 (9.1) | | 0 (0) | |  |
| Asian, Native Hawaiian, or Pacific Islander | 1 (0.5) | 3 (16.7) | 8 (4.6) | | 0 (0) | | 0 (0) | | 2 (2.0) | |  |
| Black or African American | 7 (3.3) | 0 (0) | 9 (5.1) | | 0 (0) | | 0 (0) | | 2 (2.0) | |  |
| White | 181 (84.6) | 11 (61.1) | 132 (75.4) | | 7 (46.7) | | 6 (54.6) | | 35 (35.0) | |  |
| Mixed race | 12 (5.6) | 2 (11.1) | 17 (9.7) | | 4 (26.7) | | 4 (36.4) | | 43 (43.0) | |  |
| Other | 2 (0.9) | 0 (0) | 1 (0.6) | | 3 (20.0) | | 0 (0) | | 9 (9.0) | |  |
| Prefer not to say | 8 (3.7) | 1 (5.6) | 7 (4.0) | | 1 (6.7) | | 0 (0) | | 9 (9.0) | |  |
| **Ethnicity^a^** |  |  |  | |  | |  | |  | |  |
| Hispanic | 18 (8.4) | 1 (5.6) | 8 (4.6) | | 11 (73.3) | | 8 (72.7) | | 90 (90.0) | |  |
| Non-Hispanic | 179 (83.6) | 11 (61.1) | 133 (76.0) | | 3 (20.0) | | 2 (18.2) | | 4 (4.0) | |  |
| Prefer not to say | 17 (7.9) | 6 (33.3) | 34 (19.4) | | 1 (6.7) | | 1 (9.1) | | 6 (6.0) | |  |
| **Education** |  |  |  | |  | |  | |  | |  |
| Less than high school | 8 (3.7) | 2 (11.1) | 10 (5.7) | | 2 (13.3) | | 1 (9.1) | | 9 (9.0) | |  |
| High school graduate or GED | 26 (12.2) | 3 (16.7) | 15 (8.6) | | 0 (0) | | 0 (0) | | 6 (6.0) | |  |
| Trade school or associate’s degree | 20 (9.4) | 2 (11.1) | 26 (14.9) | | 3 (20.0) | | 1 (9.1) | | 10 (10.0) | |  |
| Some college but not a graduate | 28 (13.1) | 1 (5.6) | 28 (16.0) | | 1 (6.7) | | 5 (45.5) | | 20 (20.0) | |  |
| College degree or more | 131 (61.2) | 10 (55.6) | 95 (54.3) | | 9 (60.0) | | 4 (36.4) | | 54 (54.0) | |  |
| Other | 1 (0.5) | 0 (0) | 1 (0.6) | | 0 (0) | | 0 (0) | | 1 (1.0) | |  |
| **Household income (US $)** |  |  |  | |  | |  | |  | |  |
| 0-30,000 | 25 (11.7) | 3 (16.7) | 34 (19.4) | | 8 (53.3) | | 5 (45.5) | | 42 (42.0) | |  |
| 30,001-60,000 | 41 (19.2) | 5 (27.8) | 30 (17.1) | | 3 (20.0) | | 0 (0) | | 15 (15.0) | |  |
| 60,001-100,000 | 42 (19.6) | 4 (22.2) | 31 (17.7) | | 2 (13.3) | | 2 (18.2) | | 6 (6.0) | |  |
| >$100,000 | 56 (26.2) | 3 (16.7) | 38 (21.7) | | 0 (0) | | 1 (9.1) | | 12 (12.0) | |  |
| Prefer not to say | 50 (23.4) | 3 (16.7) | 42 (24.0) | | 2 (13.3) | | 3 (27.3) | | 25 (25.0) | |  |
| **Number of people in household^a^** | | | |  | |  | |  | |  | |
| 1 | 27 (12.6) | 1 (5.6) | 40 (22.9) | | 1 (6.7) | | 3 (27.3) | | 17 (17.0) | |  |
| 2 | 139 (65.0) | 8 (44.4) | 82 (46.9) | | 7 (46.7) | | 2 (18.2) | | 42 (42.0) | |  |
| 3 | 21 (9.8) | 2 (11.1) | 20 (11.4) | | 1 (6.7) | | 2 (18.2) | | 9 (9.0) | |  |
| ≥4 | 27 (12.6) | 7 (38.9) | 33 (18.9) | | 6 (40.0) | | 4 (36.4) | | 32 (32.0) | |  |
| **Area of residence** |  |  |  | |  | |  | |  | |  |
| Urban | 64 (29.9) | 8 (44.4) | 63 (36.0) | | 12 (80.0) | | 6 (54.6) | | 84 (84.0) | |  |
| Suburban | 90 (42.1) | 5 (27.8) | 74 (42.3) | | 3 (20.0) | | 4 (36.4) | | 10 (10.0) | |  |
| Rural | 60 (28.0) | 5 (27.8) | 38 (21.7) | | 0 (0) | | 1 (9.1) | | 6 (6.0) | |  |

^a^P≤.05 comparing cancer patients and general public among English survey respondents.

Table S2. Frequency of demographic characteristics of Cook for Your Life English and Spanish female respondents who completed at least 50) of online survey by cancer survivors, primary caregivers, and general public

| Characteristic | English Respondents (n=2225), n (%) | | | Spanish Respondents | | |
| --- | --- | --- | --- | --- | --- | --- |
|  | Cancer survivors | Primary caregivers | General public | Cancer survivors | Primary caregivers | General public |
| Respondents by group | 1230 (55.3) | 200 (9.0) | 795 (35.7) | 76 (13.9) | 34 (6.2) | 435 (79.8) |
| **Region of residence^a,b,d^** |  |  |  |  |  |  |
| United States | 1014 (82.6) | 145 (72.5) | 565 (71.2) | 18.7) | 8.8) | 11.1) |
| Africa | 12 (1.0) | 1 (0.5) | 9 (1.1) | 0) | 0) | 0) |
| Asia or Pacific Islands | 32 (2.6) | 7 (3.5) | 23 (2.9) | 1.3) | 0) | 0) |
| Europe | 88 (7.2) | 22 (11.0) | 127 (16.0) | 12.0) | 8.8) | 6.5) |
| Middle East | 2 (0.2) | 1 (0.5) | 1 (0.1) | 0) | 0) | 0) |
| Canada | 70 (5.7) | 14 (7.0) | 59 (7.4) | 0) | 0) | 0.7) |
| South or Latin America | 10 (0.8) | 10 (5.0) | 10 (1.3) | 68.0) | 82.4) | 81.8) |
| **Age (years)^a,b,d^** |  |  |  |  |  |  |
| 18 - 35 | 27 (2.2) | 16 (8.0) | 85 (10.7) | 7.9) | 14.7) | 19.8) |
| 36 - 55 | 360 (29.3) | 75 (37.5) | 239 (30.1) | 35.5) | 47.1) | 43.7) |
| 56 - 65 | 355 (28.9) | 61 (30.5) | 227 (28.6) | 31.6) | 17.7) | 23.9) |
| 66 - 75 | 352 (28.6) | 40 (20.0) | 182 (22.9) | 22.4) | 20.6) | 9.7) |
| ≥76 | 136 (11.1) | 8 (4.0) | 62 (7.8) | 2.6) | 0) | 3.0) |
| **Race** |  |  |  |  |  |  |
| American Indian | 5 (0.4) | 2 (1.0) | 5 (0.6) | 2.6) | 2.9) | 0.5) |
| Asian, Native Hawaiian, or Pacific Islander | 45 (3.7) | 11 (5.5) | 29 (3.7) | 1.3) | 0) | 0.2) |
| Black or African American | 40 (3.3) | 9 (4.5) | 38 (4.8) | 2.6) | 0) | 0.7) |
| White | 1028 (83.6) | 153 (76.5) | 634 (79.8) | 39.5) | 41.2) | 37.5) |
| Mixed race | 72 (5.9) | 15 (7.5) | 52 (6.5) | 35.5) | 38.2) | 43.5) |
| Other | 13 (1.1) | 4 (2.0) | 13 (1.6) | 6.6) | 5.9) | 7.8) |
| Prefer not to say | 27 (2.2) | 6 (3.0) | 24 (3.0) | 11.8) | 11.8) | 9.9) |
| **Ethnicity^a,b^** |  |  |  |  |  |  |
| Hispanic | 61 (5.0) | 20 (10.0) | 52 (6.5) | 81.6) | 91.2) | 88.7) |
| Non-Hispanic | 1072 (87.2) | 164 (82.0) | 644 (81.0) | 10.5) | 0) | 4.8) |
| Prefer not to say | 97 (7.9) | 16 (8.0) | 99 (12.5) | 7.9) | 8.8) | 6.4) |
| **Education^b^** |  |  |  |  |  |  |
| Less than high school | 13 (1.1) | 4 (2.0) | 22 (2.8) | 5.3) | 11.8) | 6.9) |
| High school graduate or GED | 82 (6.7) | 10 (5.0) | 63 (7.9) | 17.1) | 8.8) | 10.3) |
| Trade school or associate’s degree | 105 (8.5) | 19 (9.5) | 76 (9.6) | 9.2) | 11.8) | 9.4) |
| Some college but not a graduate | 180 (14.6) | 31 (15.5) | 119 (15.0) | 17.1) | 14.7) | 18.6) |
| College degree or more | 846 (68.8) | 135 (67.5) | 512 (64.4) | 51.3) | 52.9) | 54.0) |
| Other | 4 (0.3) | 1 (0.5) | 3 (0.4) | 0) | 0) | 0.7) |
| **Household income (US $)^b^** |  |  |  |  |  |  |
| 0-30,000 | 153 (12.4) | 35 (17.5) | 130 (16.4) | 38.2) | 47.1) | 37.2) |
| 30,001-60,000 | 185 (15.0) | 33 (16.5) | 138 (17.4) | 17.1) | 14.7) | 7.6) |
| 60,001-100,000 | 250 (20.3) | 38 (19.0) | 157 (19.8) | 2.6) | 0) | 7.1) |
| >$100,000 | 341 (27.7) | 44 (22.0) | 187 (23.5) | 6.6) | 5.9) | 6.4) |
| Prefer not to say | 301 (24.5) | 50 (25.0) | 183 (23.0) | 35.5) | 32.4) | 41.6) |
| **Number of people in household^a,b^** |  |  |  |  |  |  |
| 1 | 267 (21.7) | 21 (10.5) | 187 (23.5) | 10.5) | 8.8) | 9.7) |
| 2 | 612 (49.8) | 99 (49.5) | 343 (43.1) | 29.0) | 20.6) | 25.3) |
| 3 | 161 (13.1) | 40 (20.0) | 103 (13.0) | 23.7) | 26.5) | 26.4) |
| ≥4 | 190 (15.5) | 40 (20.0) | 162 (20.4) | 36.8) | 44.1) | 38.6) |
| **Area of residence^c^** |  |  |  |  |  |  |
| Urban | 383 (31.1) | 70 (35.0) | 282 (35.5) | 68.4) | 82.4) | 77.5) |
| Suburban | 596 (48.5) | 93 (46.5) | 350 (44.0) | 25.0) | 5.9) | 15.2) |
| Rural | 251 (20.4) | 37 (18.5) | 163 (20.5) | 6.6) | 11.8) | 7.4) |

^a^P≤.05 comparing cancer patients and primary caregivers among English survey respondents.

^b^P≤.05 comparing cancer patients and general public among English survey respondents.

^c^P≤.05 comparing cancer patients and primary caregivers among Spanish survey respondents.

^d^P≤.05 comparing cancer patients and general public among Spanish survey respondents.

Table S3. Frequency of health characteristics of Cook for Your Life English and Spanish male respondents who completed at least 50% of online survey by cancer survivors, primary caregivers, and general public

|  | English Respondents (n=406), n (%) | | | Spanish Respondents (n=126), n (%) | | |
| --- | --- | --- | --- | --- | --- | --- |
|  | Cancer survivors | Primary caregivers | General public | Cancer survivors | Primary caregivers | General public |
| **Cardiometabolic conditions** |  |  |  |  |  |  |
| Chest pain | 2 (1.0) | 0 (0) | 5 (2.9) | 0 (0) | 0 (0) | 5 (5.1) |
| Diabetes or prediabetes | 35 (17.2) | 3 (21.4) | 33 (19.3) | 3 (21.4) | 2 (20.0) | 22 (22.5) |
| Hypertension | 54 (26.6) | 4 (28.6) | 57 (33.3) | 4 (28.6) | 2 (20.0) | 30 (30.6) |
| High cholesterol | 46 (22.7) | 6 (42.9) | 46 (26.9) | 2 (14.3) | 2 (20.0) | 16 (16.3) |
| Heart disease | 24 (11.8) | 3 (21.4) | 13 (7.6) | 1 (7.1) | 0 (0) | 7 (7.1) |
| Vascular disease | 13 (6.4) | 2 (14.3) | 6 (3.5) | 1 (7.1) | 0 (0) | 1 (1.0) |
| Current smoker | 9 (4.4) | 1 (7.1) | 16 (9.4) | 1 (7.1) | 2 (20.0) | 9 (9.2) |
| Drinks alcohol^a,b^ | 88 (44.2) | 6 (46.2) | 109 (65.7) | 4 (28.6) | 8 (80.0) | 49 (52.1) |
| **Number of alcoholic drinks** | n=87 | n=6 | n=108 | n=4 | n=8 | n=47 |
| 1 - 2 per week | 28 (32.2) | 2 (33.3) | 37 (34.3) | 2 (50.0) | 5 (62.5) | 30 (63.8) |
| 3 - 6 per week | 32 (36.8) | 2 (33.3) | 40 (37.0) | 2 (50.0) | 2 (25.0) | 11 (23.4) |
| Everyday | 14 (16.1) | 1 (16.7) | 10 (9.3) | 0 (0) | 0 (0) | 1 (2.1) |
| ≥2 per day | 13 (14.9) | 1 (16.7) | 21 (19.4) | 0 (0) | 1 (12.5) | 5 (10.6) |
| **Days per week eats fruit** |  |  |  |  |  |  |
| None | 6 (3.1) | 1 (7.7) | 9 (5.5) | 1 (7.1) | 0 (0) | 1 (1.1) |
| 1 - 3 days | 42 (21.5) | 4 (30.8) | 51 (30.9) | 5 (35.7) | 3 (30.0) | 27 (29.4) |
| 4 - 6 days | 59 (30.3) | 4 (30.8) | 44 (26.7) | 6 (42.9) | 3 (30.0) | 30 (32.6) |
| Everyday | 88 (45.1) | 4 (30.8) | 61 (37.0) | 2 (14.3) | 4 (40.0) | 34 (37.0) |
| **Amount of fruit when eating** | n=188 | n=12 | n=153 | n=13 | n=10 | n=89 |
| <1 cup | 121 (64.4) | 4 (33.3) | 97 (63.4) | 8 (61.5) | 2 (20.0) | 30 (33.7) |
| 1 - 2 cups | 48 (25.5) | 6 (50.0) | 41 (26.8) | 4 (30.8) | 5 (50.0) | 44 (49.4) |
| >2 cups | 19 (10.1) | 2 (16.7) | 15 (9.8) | 1 (7.7) | 3 (30.0) | 15 (16.9) |
| **Days per week eats vegetables** |  |  |  |  |  |  |
| None | 3 (1.6) | 0 (0) | 2 (1.2) | 0 (0) | 0 (0) | 0 (0) |
| 1 - 3 days | 26 (13.4) | 3 (23.1) | 21 (13.0) | 4 (28.6) | 3 (30.0) | 29 (32.2) |
| 4 - 6 days | 70 (36.1) | 5 (38.5) | 59 (36.4) | 6 (42.9) | 3 (30.0) | 37 (41.1) |
| Everyday | 95 (49.0) | 5 (38.5) | 80 (49.4) | 4 (28.6) | 4 (40.0) | 24 (26.7) |
| **Amount of vegetables when eating^a^** | n=184 | n=13 | n=150 | n=14 | n=10 | n=86 |
| <1 cup | 125 (67.9) | 5 (38.5) | 78 (52.0) | 10 (71.4) | 2 (20.0) | 36 (41.9) |
| 1 - 2 cups | 50 (27.2) | 6 (46.2) | 51 (34.0) | 3 (21.4) | 5 (50.0) | 35 (40.7) |
| >2 cups | 9 (4.9) | 2 (15.4) | 21 (14.0) | 1 (7.1) | 3 (30.0) | 15 (17.4) |
| **Days per week of MVPA^c^** |  |  |  |  |  |  |
| None | 37 (19.9) | 3 (23.1) | 19 (12.6) | 3 (21.4) | 1 (10.0) | 19 (22.1) |
| 1 - 3 days | 65 (35.0) | 6 (46.2) | 55 (36.4) | 5 (35.7) | 5 (50.0) | 37 (43.0) |
| 4 - 6 days | 59 (31.7) | 4 (30.8) | 53 (35.1) | 3 (21.4) | 4 (40.0) | 23 (26.7) |
| Everyday | 25 (13.4) | 0 (0) | 24 (15.9) | 3 (21.4) | 0 (0) | 7 (8.1) |
| **Minutes per day in MVPA^c^** |  |  |  |  |  |  |
| 0 - <10 | 40 (21.5) | 2 (15.4) | 21 (13.9) | 4 (28.6) | 0 (0) | 21 (24.4) |
| 10 - <30 | 54 (29.0) | 6 (46.2) | 35 (23.2) | 6 942.9) | 3 (30.0) | 22 (25.6) |
| 30 - <40 | 36 (19.4) | 3 (23.1) | 31 (20.5) | 0 (0) | 2 (20.0) | 17 (19.8) |
| 40 - <60 | 34 (18.3) | 2 (15.4) | 38 (25.2) | 4 (28.6) | 4 (40.0) | 17 (19.8) |
| ≥60 | 22 (11.8) | 0 (0) | 26 (17.2) | 0 (0) | 1 (10.0) | 9 (10.5) |

^a^P≤.05 comparing cancer patients and general public among English survey respondents.

^b^P≤.05 comparing cancer patients and primary caregivers among Spanish survey respondents.

^c^MVPA: moderate-to-vigorous physical activity

Table S4. Frequency of health characteristics of Cook for Your Life English and Spanish female respondents who completed at least 50) of online survey by cancer survivors, primary caregivers, and general public

|  | English respondents (n=2225), n (%) | | | | | Spanish Respondents (n=545), n (%) | | | |
| --- | --- | --- | --- | --- | --- | --- | --- | --- | --- |
|  | Cancer survivors | Primary caregivers | | General public | | Cancer survivors | Primary caregivers | | General public |
| **Cardiometabolic conditions** | | |  | |  |  |  |  | |
| Chest pain | 26 (2.2) | 7 (3.7) | | 17 (2.2) | | 2 (2.7) | 1 (3.2) | | 14 (3.3) |
| Diabetes or prediabetes | 160 (13.6) | 25 (13.0) | | 119 (15.5) | | 13 (17.6) | 4 (12.9) | | 54 (12.9) |
| Hypertension^c^ | 280 (23.7) | 50 (26.0) | | 189 (24.6) | | 22 (29.7) | 7 (22.6) | | 83 (19.8) |
| High cholesterol | 241 (20.4) | 38 (19.8) | | 168 (21.8) | | 16 (21.6) | 12 (38.7) | | 73 (17.4) |
| Heart disease | 40 (3.4) | 7 (3.7) | | 31 (4.0) | | 1 (1.4) | 3 (9.7) | | 11 (2.6) |
| Vascular disease | 23 (2.0) | 5 (2.6) | | 20 (2.6) | | 1 (1.4) | 1 (3.2) | | 5 (1.2) |
| Current smoker^a,b^ | 26 (2.2) | 10 (5.2) | | 46 (6.0) | | 7 (9.5) | 4 (12.9) | | 45 (10.7) |
| Drinks alcohol^b,c^ | 533 (46.0) | 82 (45.8) | | 425 (57.7) | | 18 (24.7) | 11 (36.7) | | 150 (36.9) |
| **Number of alcoholic drinks^a,b^** | n=528 | n=80 | | n=421 | | n=18 | n=11 | | n=148 |
| 1 - 2 per week | 289 (54.7) | 41 (51.3) | | 165 (39.2) | | 12 (66.7) | 7 (63.6) | | 103 (69.6) |
| 3 - 6 per week | 159 (30.1) | 21 (26.3) | | 145 (34.4) | | 4 (22.2) | 3 (27.3) | | 32 (21.6) |
| Everyday | 42 (8.0) | 3 (3.8) | | 45 (10.7) | | 1 (5.6) | 1 (9.1) | | 8 (5.4) |
| ≥2 per day | 38 (7.2) | 15 (18.8) | | 66 (15.7) | | 1 (5.6) | 0 (0) | | 5 (3.4) |
| **Days per week eats fruit^a,b^** |  |  | |  | |  |  | |  |
| None | 29 (2.5) | 5 (2.8) | | 28 (3.8) | | 2 (2.7) | 1 (3.3) | | 15 (3.7) |
| 1 - 3 days | 209 (18.1) | 48 (27.3) | | 174 (23.9) | | 19 (26.0) | 7 (23.3) | | 141 (34.9) |
| 4 - 6 days | 355 (30.8) | 60 (34.1) | | 230 (31.6) | | 25 (34.3) | 14 (46.7) | | 116 (28.7) |
| Everyday | 559 (48.5) | 63 (35.8) | | 297 (40.7) | | 27 (37.0) | 8 (26.7) | | 132 (32.7) |
| **Amount of fruit when eating** | n=1117 | n=170 | | n=694 | | n=70 | n=29 | | n=387 |
| <1 cup | 726 (65.0) | 112 (65.9) | | 442 (63.7) | | 42 (60.0) | 15 (51.7) | | 201 (51.9) |
| 1 - 2 cups | 302 (27.0) | 43 (25.3) | | 193 (27.8) | | 19 (27.1) | 10 (34.5) | | 129 (33.3) |
| >2 cups | 89 (8.0) | 15 (8.8) | | 59 (8.5) | | 9 (12.9) | 4 (13.8) | | 57 (14.7) |
| **Days per week eats vegetables** | |  | |  | |  |  | |  |
| None | 9 (0.8) | 0 (0) | | 7 (1.0) | | 2 (2.8) | 0 (0) | | 5 (1.2) |
| 1 - 3 days | 110 (9.6) | 16 (9.1) | | 81 (11.2) | | 18 (25.0) | 7 (23.3) | | 92 (22.9) |
| 4 - 6 days | 343 (29.9) | 65 (37.1) | | 223 (30.9) | | 26 (36.1) | 12 (40.0) | | 150 (37.3) |
| Everyday | 684 (59.7) | 94 (53.7) | | 411 (56.9) | | 26 (36.1) | 11 (36.7) | | 155 (38.6) |
| **Amount of vegetables when eating** | n=1123 | n=169 | | n=687 | | n=70 | n=30 | | n=380 |
| <1 cup | 641 (57.1) | 89 (52.7) | | 357 (52.0) | | 38 (54.3) | 21 (70.0) | | 185 (48.7) |
| 1 - 2 cups | 382 (34.0) | 59 (34.9) | | 256 (37.3) | | 24 (34.3) | 8 (26.7) | | 136 (35.8) |
| >2 cups | 100 (8.9) | 21 (12.4) | | 74 (10.8) | | 8 (11.4) | 1 (3.3) | | 59 (15.5) |
| **Days per week of MVPA^d^** |  |  | |  | |  |  | |  |
| None | 167 (14.8) | 25 (14.8) | | 102 (14.7) | | 22 (31.0) | 6 (20.0) | | 105 (27.3) |
| 1 - 3 days | 406 (35.9) | 67 (39.6) | | 258 (37.2) | | 30 (42.3) | 16 (53.3) | | 169 (43.9) |
| 4 - 6 days | 424 (37.5) | 58 (34.3) | | 246 (35.5) | | 15 (21.1) | 7 (23.3) | | 84 (21.8) |
| Everyday | 135 (11.9) | 19 (11.2) | | 87 (12.6) | | 4 (5.6) | 1 (3.3) | | 27 (7.0) |
| **Minutes per day in MVPA^d^** |  |  | |  | |  |  | |  |
| 0 - <10 | 177 (15.6) | 29 (17.2) | | 110 (15.9) | | 20 (28.2) | 6 (20.0) | | 110 (28.6) |
| 10 - <30 | 295 (26.1) | 49 (29.0) | | 176 (25.4) | | 19 (26.8) | 13 (43.3) | | 86 (22.3) |
| 30 - <40 | 259 (22.9) | 34 (20.1) | | 152 (21.9) | | 17 (23.9) | 4 (13.3) | | 59 (15.3) |
| 40 - <60 | 282 (24.9) | 39 (23.1) | | 169 (24.4) | | 10 (14.1) | 5 (16.7) | | 83 (21.6) |
| ≥60 | 119 (10.5) | 18 (10.7) | | 86 (12.4) | | 5 (7.0) | 2 (6.7) | | 47 (12.2) |

^a^P≤.05 comparing cancer patients and primary caregivers among English survey respondents.

^b^P≤.05 comparing cancer patients and general public among English survey respondents.

^c^P≤.05 comparing cancer patients and general public among Spanish survey respondents.

^d^MVPA: moderate-to-vigorous physical activity.
